# 3-(Di­phenyl­amino)­isobenzo­furan-1(3*H*)-one

**DOI:** 10.1107/S1600536814006266

**Published:** 2014-03-29

**Authors:** Rodolfo Moreno-Fuquen, Juan C. Castillo, Rodrigo Abonia, Javier Ellena, Juan C. Tenorio

**Affiliations:** a Departamento de Química - Facultad de Ciencias Naturales y Exactas, Universidad del Valle, Apartado 25360, Santiago de Cali, Colombia; b Instituto de Física de São Carlos, IFSC, Universidade de São Paulo, USP, São Carlos, SP, Brazil

## Abstract

In the title isobenzo­furan­one derivative, C_20_H_15_NO_2_, the planar fused-ring system (r.m.s. deviation for the 10 fitted atoms = 0.031 Å) forms dihedral angles of 63.58 (6) and 63.17 (8)° with the N-bound phenyl rings; the dihedral angle between the planes of these phenyl rings is 85.92 (7)°. In the crystal, mol­ecules are linked by weak C—H⋯O inter­actions, involving both O atoms, forming helical supra­molecular chains along [001].

## Related literature   

For biological and pharmacological properties of isobenzo­furan­ones, see: Anderson *et al.* (2005 ▶); Malpani *et al.* (2013 ▶); Shode *et al.* (2002 ▶); Yoganathan *et al.* (2003 ▶). For the synthesis of diverse amino derivatives, see: Abonia *et al.* (2010 ▶, 2013 ▶); Moreno-Fuquen *et al.* (2013 ▶). For similar structures, see: Mendenhall *et al.* (2003 ▶); Reynolds & Scaringe (1982 ▶).
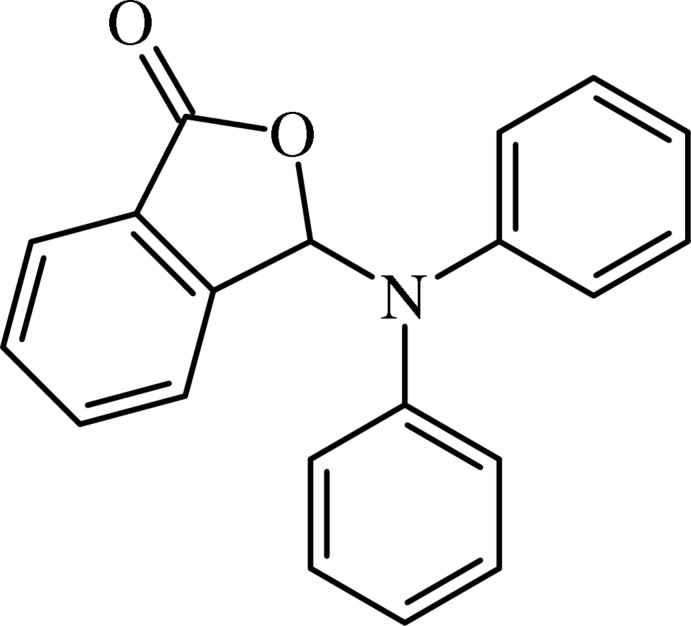


## Experimental   

### 

#### Crystal data  

C_20_H_15_NO_2_
*M* *_r_* = 301.33
Orthorhombic, 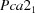

*a* = 19.1440 (13) Å
*b* = 8.9363 (6) Å
*c* = 9.1111 (3) Å
*V* = 1558.70 (16) Å^3^
*Z* = 4
Mo *K*α radiation
μ = 0.08 mm^−1^
*T* = 295 K
0.56 × 0.37 × 0.19 mm

#### Data collection  

Nonius KappaCCD diffractometer
3011 measured reflections
1684 independent reflections
1366 reflections with *I* > 2σ(*I*)
*R*_int_ = 0.018

#### Refinement  

*R*[*F* ^2^ > 2σ(*F* ^2^)] = 0.041
*wR*(*F* ^2^) = 0.106
*S* = 1.09
1684 reflections
213 parameters
1 restraint
H atoms treated by a mixture of independent and constrained refinement
Δρ_max_ = 0.15 e Å^−3^
Δρ_min_ = −0.15 e Å^−3^

### 

Data collection: *COLLECT* (Nonius, 2000 ▶); cell refinement: *SCALEPACK* (Otwinowski & Minor, 1997 ▶); data reduction: *DENZO* (Otwinowski & Minor, 1997 ▶) and *SCALEPACK*; program(s) used to solve structure: *SHELXS97* (Sheldrick, 2008 ▶); program(s) used to refine structure: *SHELXL97* (Sheldrick, 2008 ▶); molecular graphics: *ORTEP-3 for Windows* (Farrugia, 2012 ▶) and *Mercury* (Macrae *et al.*, 2006 ▶); software used to prepare material for publication: *WinGX* (Farrugia, 2012 ▶).

## Supplementary Material

Crystal structure: contains datablock(s) I, global. DOI: 10.1107/S1600536814006266/tk5299sup1.cif

Structure factors: contains datablock(s) I. DOI: 10.1107/S1600536814006266/tk5299Isup2.hkl

Supporting information file. DOI: 10.1107/S1600536814006266/tk5299Isup3.cml

CCDC reference: 992870

Additional supporting information:  crystallographic information; 3D view; checkCIF report

## Figures and Tables

**Table 1:** Hydrogen-bond geometry (Å, °)

*D*—H⋯*A*	*D*—H	H⋯*A*	*D*⋯*A*	*D*—H⋯*A*
C3—H3⋯O2^i^	0.93	2.70	3.413 (3)	135
C1—H1⋯O1^i^	1.03 (3)	2.36 (3)	3.307 (3)	153 (2)

Symmetry code: (i) 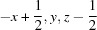.

**Fig. 1.**
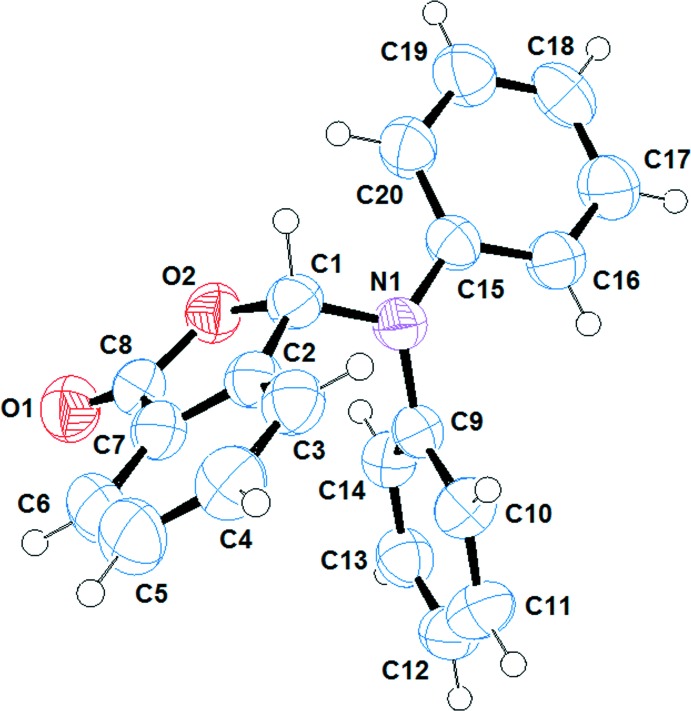
Molecular conformation and atom numbering scheme for (I) with displacement ellipsoids drawn at the 50% probability level. H atoms are shown as spheres of arbitrary radius.

**Fig. 2.**
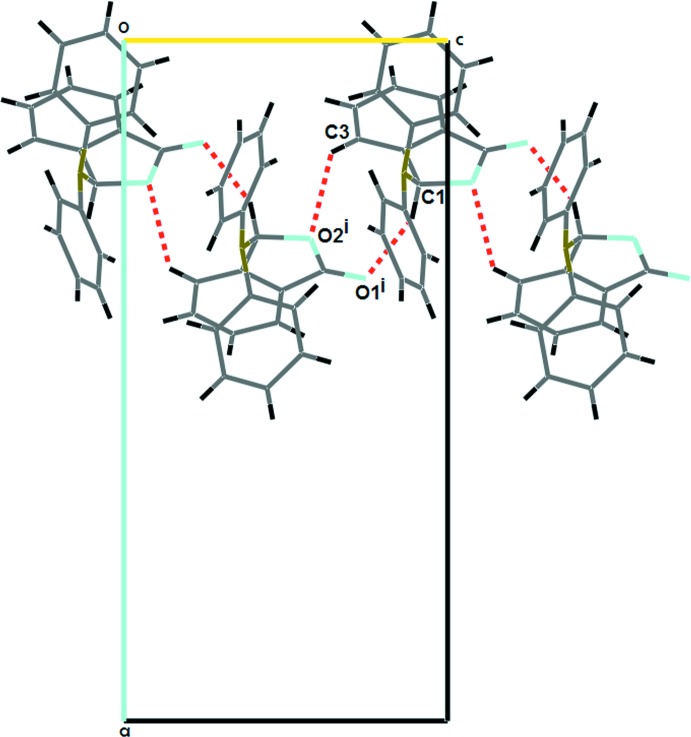
Part of the crystal structure of (I), showing the formation of helical chains running along [001]. Symmetry code: (i) -x+1/2, +y, +z-1/2.

## supplementary crystallographic information

### 2.1. Synthesis and crystallization

Reagents and solvents for the synthesis were obtained from the Aldrich Chemical Co., and were used without additional purification. A 5 mL pyrex test tube was charged with a mixture of di­phenyl­amine (102 mg, 0.60 mmol) and 2-formyl­benzoic acid (90 mg, 0.60 mmol) without solvent. The mixture was heated in an oil bath at 120 °C for 1 h until the starting materials were not longer detected by thin-layer chromatography. The solid formed was removed and washed with cold ethanol (1 mL). White crystals of (I) were grown by slow evaporation, under ambient conditions, from its solution in ethanol [92% yield, M.p.: 396 (1) K].

### 2.2. Refinement

All H-atoms, except H1, were positioned at geometrically idealized positions, C—H = 0.93 Å, and they were refined using a riding model approximation with *U*_iso_(H) = 1.2*U*_eq_(parent atom). Atom H1 was found from the Fourier difference map and its coordinates were freely refined. In the absence of significant anomalous scattering, Friedel pairs were merged.

### 3. Results and discussion

Isobenzo­furan­ones are an important class of synthetic and naturally occurring products exhibiting diverse biological and pharmacological properties. Some of them appear forming part of the structure of natural products such as fuscinarin (anti-HIV properties) (Yoganathan *et al.*, 2003), typhaphthalide (phenolic compound isolated from Typha capensis) (Shode *et al.*, 2002), noscapine (anti­tussive and anti-tumor properties) (Anderson *et al.*, 2005), and synthetic compounds like some spiro­lactones (inhibitors of the influenza virus type B) (Malpani *et al.*, 2013). Continuing with our current studies on the use of imines and imminium ions for the synthesis of diverse amino-derivatives of synthetic and biological inter­est (Abonia *et al.*, 2010; Abonia *et al.*, 2013; Moreno-Fuquen *et al.*, 2013), 3-di­phenyl­amino­isobenzo­furan-1(3*H*)-one, (I), was obtained from the reaction of 2-formyl­benzoic acid and di­phenyl­amine through an imminium ion inter­mediate. The molecular structure of (I) is shown in Fig. 1. Taking the plane of the phthalide lactone C1—C8(=O1)—O2 (Mendenhall *et al.*, 2003) as a point of reference, the title compound represents the first structure reported with ligands from C1. The bond lengths reported in the phthalide lactone (Reynolds & Scaringe, 1982) are very similar to those presented in (I). In the present molecule, rings A (C9—C14), B (C1—C8—O2) and C (C15—C20) are planar and show dihedral angles between them: A/B = 63.58 (6)°, C/B= 63.17 (8)° and A/C= 85.92 (7)°. In the crystal, the molecules are linked by weak C—H···O inter­actions, forming eight-membered {···HC_3_H···OCO} synthons, leading to a chain along [001], Table 1 and Fig. 2.

### Crystal data

**Table:**

C_20_H_15_NO_2_	*D*_x_ = 1.284 Mg m^−^^3^
*M**_r_* = 301.33	Melting point: 396(1) K
Orthorhombic, *P**c**a*2_1_	Mo *K*α radiation, λ = 0.71073 Å
Hall symbol: P 2c -2ac	Cell parameters from 2966 reflections
*a* = 19.1440 (13) Å	θ = 3.1–26.4°
*b* = 8.9363 (6) Å	µ = 0.08 mm^−^^1^
*c* = 9.1111 (3) Å	*T* = 295 K
*V* = 1558.70 (16) Å^3^	Block, white
*Z* = 4	0.56 × 0.37 × 0.19 mm
*F*(000) = 632	

### Data collection

**Table:**

Nonius KappaCCD diffractometer	1366 reflections with *I* > 2σ(*I*)
Radiation source: fine-focus sealed tube	*R*_int_ = 0.018
Graphite monochromator	θ_max_ = 26.4°, θ_min_ = 3.1°
CCD rotation images, thick slices scans	*h* = −23→23
3011 measured reflections	*k* = −11→11
1684 independent reflections	*l* = −11→11

### Refinement

**Table:**

Refinement on *F*^2^	Secondary atom site location: difference Fourier map
Least-squares matrix: full	Hydrogen site location: inferred from neighbouring sites
*R*[*F*^2^ > 2σ(*F*^2^)] = 0.041	H atoms treated by a mixture of independent and constrained refinement
*wR*(*F*^2^) = 0.106	*w* = 1/[σ^2^(*F*_o_^2^) + (0.0695*P*)^2^ + 0.0242*P*] where *P* = (*F*_o_^2^ + 2*F*_c_^2^)/3
*S* = 1.09	(Δ/σ)_max_ < 0.001
1684 reflections	Δρ_max_ = 0.15 e Å^−^^3^
213 parameters	Δρ_min_ = −0.15 e Å^−^^3^
1 restraint	Extinction correction: *SHELXL97* (Sheldrick, 2008), Fc^*^=kFc[1+0.001xFc^2^λ^3^/sin(2θ)]^-1/4^
Primary atom site location: structure-invariant direct methods	Extinction coefficient: 0.042 (9)

### Special details

**Table:**

Geometry. All esds (except the esd in the dihedral angle between two l.s. planes) are estimated using the full covariance matrix. The cell esds are taken into account individually in the estimation of esds in distances, angles and torsion angles; correlations between esds in cell parameters are only used when they are defined by crystal symmetry. An approximate (isotropic) treatment of cell esds is used for estimating esds involving l.s. planes.
Refinement. Refinement of *F*^2^ against ALL reflections. The weighted *R*-factor *wR* and goodness of fit *S* are based on *F*^2^, conventional *R*-factors *R* are based on *F*, with *F* set to zero for negative *F*^2^. The threshold expression of *F*^2^ > σ(*F*^2^) is used only for calculating *R*-factors(gt) *etc*. and is not relevant to the choice of reflections for refinement. *R*-factors based on *F*^2^ are statistically about twice as large as those based on *F*, and *R*- factors based on ALL data will be even larger.

### Fractional atomic coordinates and isotropic or equivalent isotropic displacement parameters (Å^2^)

**Table:**

	*x*	*y*	*z*	*U*_iso_*/*U*_eq_	
O1	0.34995 (11)	1.2679 (2)	0.7466 (2)	0.0689 (6)	
O2	0.29111 (9)	1.13804 (19)	0.5762 (2)	0.0578 (5)	
N1	0.30495 (10)	0.9786 (2)	0.3646 (3)	0.0509 (5)	
C1	0.28865 (13)	1.1262 (3)	0.4127 (3)	0.0517 (6)	
C2	0.33858 (12)	1.2458 (3)	0.3647 (3)	0.0513 (6)	
C3	0.35485 (15)	1.2966 (3)	0.2247 (3)	0.0580 (6)	
H3	0.3353	1.2529	0.1417	0.070*	
C4	0.40139 (16)	1.4151 (3)	0.2135 (4)	0.0672 (7)	
H4	0.4129	1.4516	0.1210	0.081*	
C5	0.43103 (17)	1.4803 (3)	0.3362 (4)	0.0761 (9)	
H5	0.4626	1.5586	0.3248	0.091*	
C6	0.41460 (15)	1.4311 (3)	0.4744 (4)	0.0705 (8)	
H6	0.4343	1.4751	0.5572	0.085*	
C7	0.36754 (13)	1.3130 (3)	0.4872 (3)	0.0549 (6)	
C8	0.33827 (13)	1.2427 (3)	0.6184 (3)	0.0544 (6)	
C9	0.37543 (12)	0.9290 (2)	0.3913 (3)	0.0487 (6)	
C10	0.42604 (13)	0.9458 (3)	0.2845 (3)	0.0572 (6)	
H10	0.4143	0.9877	0.1944	0.069*	
C11	0.49369 (14)	0.9008 (3)	0.3103 (3)	0.0649 (7)	
H11	0.5275	0.9128	0.2380	0.078*	
C12	0.51118 (14)	0.8386 (3)	0.4422 (3)	0.0627 (7)	
H12	0.5569	0.8083	0.4596	0.075*	
C13	0.46114 (13)	0.8206 (3)	0.5498 (3)	0.0611 (6)	
H13	0.4732	0.7782	0.6395	0.073*	
C14	0.39338 (13)	0.8653 (3)	0.5245 (3)	0.0556 (7)	
H14	0.3597	0.8526	0.5969	0.067*	
C15	0.25151 (13)	0.8676 (2)	0.3667 (3)	0.0515 (6)	
C16	0.26525 (14)	0.7304 (3)	0.3021 (4)	0.0699 (8)	
H16	0.3089	0.7130	0.2607	0.084*	
C17	0.21563 (16)	0.6196 (3)	0.2981 (5)	0.0791 (9)	
H17	0.2261	0.5282	0.2547	0.095*	
C18	0.15013 (16)	0.6429 (3)	0.3583 (4)	0.0710 (8)	
H18	0.1167	0.5675	0.3569	0.085*	
C19	0.13553 (17)	0.7796 (4)	0.4199 (4)	0.0743 (8)	
H19	0.0912	0.7978	0.4576	0.089*	
C20	0.18593 (15)	0.8909 (3)	0.4269 (3)	0.0663 (7)	
H20	0.1757	0.9816	0.4721	0.080*	
H1	0.2377 (16)	1.155 (3)	0.390 (3)	0.062 (8)*	

### Atomic displacement parameters (Å^2^)

**Table:**

	*U*^11^	*U*^22^	*U*^33^	*U*^12^	*U*^13^	*U*^23^
O1	0.0755 (12)	0.0675 (12)	0.0638 (13)	0.0031 (9)	−0.0014 (9)	−0.0069 (9)
O2	0.0599 (11)	0.0522 (10)	0.0612 (11)	−0.0026 (8)	0.0074 (8)	−0.0047 (8)
N1	0.0455 (10)	0.0432 (10)	0.0639 (11)	−0.0007 (8)	−0.0019 (9)	−0.0033 (9)
C1	0.0475 (15)	0.0471 (14)	0.0606 (15)	0.0006 (10)	0.0022 (10)	0.0001 (11)
C2	0.0478 (13)	0.0414 (12)	0.0647 (15)	0.0025 (9)	0.0014 (11)	−0.0001 (11)
C3	0.0596 (15)	0.0501 (15)	0.0644 (16)	0.0045 (12)	0.0002 (11)	0.0004 (12)
C4	0.0746 (17)	0.0537 (15)	0.0734 (18)	−0.0006 (13)	0.0104 (14)	0.0085 (14)
C5	0.083 (2)	0.0531 (15)	0.093 (2)	−0.0173 (14)	0.0150 (16)	−0.0035 (15)
C6	0.0754 (18)	0.0539 (15)	0.082 (2)	−0.0149 (14)	0.0009 (16)	−0.0087 (14)
C7	0.0539 (13)	0.0439 (13)	0.0669 (14)	0.0031 (11)	0.0010 (12)	−0.0045 (12)
C8	0.0522 (13)	0.0489 (14)	0.0623 (16)	0.0059 (11)	0.0001 (11)	−0.0064 (12)
C9	0.0484 (13)	0.0420 (12)	0.0556 (14)	−0.0046 (10)	0.0000 (10)	−0.0038 (10)
C10	0.0593 (14)	0.0572 (14)	0.0550 (13)	0.0032 (12)	0.0052 (12)	0.0058 (12)
C11	0.0542 (15)	0.0651 (16)	0.0755 (17)	0.0043 (12)	0.0137 (13)	0.0084 (14)
C12	0.0505 (14)	0.0598 (15)	0.0777 (18)	0.0022 (12)	−0.0056 (12)	−0.0021 (13)
C13	0.0650 (16)	0.0601 (15)	0.0581 (14)	0.0026 (13)	−0.0091 (13)	−0.0005 (13)
C14	0.0578 (15)	0.0550 (15)	0.0539 (16)	−0.0006 (12)	0.0014 (11)	−0.0014 (10)
C15	0.0529 (13)	0.0477 (12)	0.0540 (12)	−0.0047 (11)	−0.0036 (11)	0.0012 (10)
C16	0.0576 (16)	0.0536 (14)	0.098 (2)	−0.0017 (12)	−0.0010 (15)	−0.0138 (16)
C17	0.074 (2)	0.0509 (15)	0.112 (3)	−0.0070 (13)	−0.0064 (18)	−0.0137 (18)
C18	0.076 (2)	0.0639 (17)	0.0731 (17)	−0.0251 (14)	−0.0109 (15)	0.0048 (15)
C19	0.0645 (16)	0.087 (2)	0.0717 (17)	−0.0225 (15)	0.0127 (14)	−0.0074 (16)
C20	0.0624 (17)	0.0656 (16)	0.0709 (16)	−0.0131 (13)	0.0122 (13)	−0.0122 (14)

### Geometric parameters (Å, º)

**Table:**

O1—C8	1.210 (4)	C10—C11	1.376 (4)
O2—C8	1.355 (3)	C10—H10	0.9300
O2—C1	1.494 (3)	C11—C12	1.366 (4)
N1—C15	1.425 (3)	C11—H11	0.9300
N1—C1	1.425 (3)	C12—C13	1.380 (4)
N1—C9	1.441 (3)	C12—H12	0.9300
C1—C2	1.499 (3)	C13—C14	1.377 (4)
C1—H1	1.03 (3)	C13—H13	0.9300
C2—C7	1.383 (4)	C14—H14	0.9300
C2—C3	1.389 (4)	C15—C16	1.385 (4)
C3—C4	1.388 (4)	C15—C20	1.386 (4)
C3—H3	0.9300	C16—C17	1.372 (4)
C4—C5	1.383 (5)	C16—H16	0.9300
C4—H4	0.9300	C17—C18	1.384 (5)
C5—C6	1.371 (5)	C17—H17	0.9300
C5—H5	0.9300	C18—C19	1.373 (5)
C6—C7	1.392 (4)	C18—H18	0.9300
C6—H6	0.9300	C19—C20	1.387 (4)
C7—C8	1.462 (4)	C19—H19	0.9300
C9—C10	1.382 (3)	C20—H20	0.9300
C9—C14	1.384 (3)		
			
C8—O2—C1	110.6 (2)	C11—C10—C9	120.5 (3)
C15—N1—C1	118.89 (19)	C11—C10—H10	119.8
C15—N1—C9	117.15 (18)	C9—C10—H10	119.8
C1—N1—C9	115.92 (19)	C12—C11—C10	120.0 (2)
N1—C1—O2	111.4 (2)	C12—C11—H11	120.0
N1—C1—C2	115.5 (2)	C10—C11—H11	120.0
O2—C1—C2	102.75 (19)	C11—C12—C13	120.2 (2)
N1—C1—H1	112.2 (15)	C11—C12—H12	119.9
O2—C1—H1	102.2 (17)	C13—C12—H12	119.9
C2—C1—H1	111.6 (15)	C14—C13—C12	120.1 (3)
C7—C2—C3	120.6 (2)	C14—C13—H13	120.0
C7—C2—C1	109.2 (2)	C12—C13—H13	120.0
C3—C2—C1	130.1 (2)	C13—C14—C9	120.0 (2)
C4—C3—C2	117.5 (3)	C13—C14—H14	120.0
C4—C3—H3	121.3	C9—C14—H14	120.0
C2—C3—H3	121.3	C16—C15—C20	118.3 (2)
C5—C4—C3	121.7 (3)	C16—C15—N1	118.3 (2)
C5—C4—H4	119.2	C20—C15—N1	123.4 (2)
C3—C4—H4	119.2	C17—C16—C15	121.2 (3)
C6—C5—C4	120.9 (3)	C17—C16—H16	119.4
C6—C5—H5	119.5	C15—C16—H16	119.4
C4—C5—H5	119.5	C16—C17—C18	120.6 (3)
C5—C6—C7	117.9 (3)	C16—C17—H17	119.7
C5—C6—H6	121.0	C18—C17—H17	119.7
C7—C6—H6	121.0	C19—C18—C17	118.7 (3)
C2—C7—C6	121.4 (3)	C19—C18—H18	120.7
C2—C7—C8	108.6 (2)	C17—C18—H18	120.7
C6—C7—C8	129.9 (3)	C18—C19—C20	121.0 (3)
O1—C8—O2	121.7 (2)	C18—C19—H19	119.5
O1—C8—C7	129.6 (2)	C20—C19—H19	119.5
O2—C8—C7	108.6 (2)	C19—C20—C15	120.3 (3)
C10—C9—C14	119.2 (2)	C19—C20—H20	119.9
C10—C9—N1	120.3 (2)	C15—C20—H20	119.9
C14—C9—N1	120.5 (2)		
			
C15—N1—C1—O2	81.6 (3)	C6—C7—C8—O2	176.1 (3)
C9—N1—C1—O2	−66.3 (3)	C15—N1—C9—C10	117.8 (2)
C15—N1—C1—C2	−161.6 (2)	C1—N1—C9—C10	−93.7 (3)
C9—N1—C1—C2	50.4 (3)	C15—N1—C9—C14	−62.7 (3)
C8—O2—C1—N1	121.2 (2)	C1—N1—C9—C14	85.9 (3)
C8—O2—C1—C2	−3.1 (3)	C14—C9—C10—C11	−0.6 (4)
N1—C1—C2—C7	−119.6 (2)	N1—C9—C10—C11	179.0 (2)
O2—C1—C2—C7	2.0 (3)	C9—C10—C11—C12	0.2 (4)
N1—C1—C2—C3	63.7 (4)	C10—C11—C12—C13	0.0 (4)
O2—C1—C2—C3	−174.8 (3)	C11—C12—C13—C14	0.0 (4)
C7—C2—C3—C4	0.7 (4)	C12—C13—C14—C9	−0.3 (4)
C1—C2—C3—C4	177.1 (3)	C10—C9—C14—C13	0.6 (4)
C2—C3—C4—C5	0.4 (4)	N1—C9—C14—C13	−178.9 (2)
C3—C4—C5—C6	−0.9 (5)	C1—N1—C15—C16	171.7 (3)
C4—C5—C6—C7	0.4 (5)	C9—N1—C15—C16	−40.7 (3)
C3—C2—C7—C6	−1.2 (4)	C1—N1—C15—C20	−6.7 (4)
C1—C2—C7—C6	−178.3 (2)	C9—N1—C15—C20	140.9 (3)
C3—C2—C7—C8	176.8 (2)	C20—C15—C16—C17	−0.4 (5)
C1—C2—C7—C8	−0.3 (3)	N1—C15—C16—C17	−178.8 (3)
C5—C6—C7—C2	0.7 (4)	C15—C16—C17—C18	0.4 (5)
C5—C6—C7—C8	−176.9 (3)	C16—C17—C18—C19	1.0 (5)
C1—O2—C8—O1	−178.2 (2)	C17—C18—C19—C20	−2.3 (5)
C1—O2—C8—C7	3.0 (3)	C18—C19—C20—C15	2.3 (5)
C2—C7—C8—O1	179.6 (3)	C16—C15—C20—C19	−0.9 (4)
C6—C7—C8—O1	−2.6 (5)	N1—C15—C20—C19	177.5 (3)
C2—C7—C8—O2	−1.7 (3)		

### Hydrogen-bond geometry (Å, º)

**Table:**

*D*—H···*A*	*D*—H	H···*A*	*D*···*A*	*D*—H···*A*
C3—H3···O2^i^	0.93	2.70	3.413 (3)	135
C1—H1···O1^i^	1.03 (3)	2.36 (3)	3.307 (3)	153 (2)

Symmetry code: (i) −*x*+1/2, *y*, *z*−1/2.

## References

[bb1] Abonia, R., Castillo, J., Insuasty, B., Quiroga, J., Nogueras, M. & Cobo, J. (2010). *Eur. J. Org. Chem* pp. 6454–6463.

[bb2] Abonia, R., Castillo, J., Insuasty, B., Quiroga, J., Nogueras, M. & Cobo, J. (2013). *Comb. Sci* **15**, 2–9. 10.1021/co300105t 23167924

[bb3] Anderson, J. T., Ting, A. E., Boozer, S., Brunden, K. R., Crumrine, C., Danzig, J., Dent, T., Faga, L., Harrington, J. J., Hodnick, W. F., Murphy, S. M., Pawlowski, G., Perry, R., Raber, A., Rundlett, S. E., Stricker-Krongrad, A., Wang, J. & Bennani, Y. L. (2005). *J. Med. Chem.* **48**, 7096–7098. 10.1021/jm050674q 16279766

[bb4] Farrugia, L. J. (2012). *J. Appl. Cryst.* **45**, 849–854.

[bb5] Macrae, C. F., Edgington, P. R., McCabe, P., Pidcock, E., Shields, G. P., Taylor, R., Towler, M. & van de Streek, J. (2006). *J. Appl. Cryst.* **39**, 453–457.

[bb6] Malpani, Y., Achary, R., Kim, S. Y., Jeong, H. C., Kim, P., Han, S. B., Kim, M., Lee, C.-K., Kim, J. N. & Jung, Y.-S. (2013). *Eur. J. Med. Chem.* **62**, 534–544. 10.1016/j.ejmech.2013.01.015 23419738

[bb7] Mendenhall, G. D., Luck, R. L., Bohn, R. K. & Castejon, H. J. (2003). *J. Mol. Struct* **645**, 249–258.

[bb8] Moreno-Fuquen, R., Castillo, J. C., Abonia, R., Ellena, J. & Tenorio, J. C. (2013). *Acta Cryst.* E**69**, o1181–o1182. 10.1107/S1600536813017479 PMC3770437 24046722

[bb9] Nonius (2000). *COLLECT* Nonius BV, Delft, The Netherlands.

[bb10] Otwinowski, Z. & Minor, W. (1997). *Methods in Enzymology*, Vol. 276, *Macromolecular Crystallography*, Part A, edited by C. W. Carter Jr & R. M. Sweet, pp. 307–326. New York: Academic Press.

[bb11] Reynolds, S. L. & Scaringe, R. P. (1982). *Cryst. Struct. Commun* **11**, 1129–1134.

[bb12] Sheldrick, G. M. (2008). *Acta Cryst.* A**64**, 112–122. 10.1107/S0108767307043930 18156677

[bb13] Shode, F. O., Mahomed, A. S. & Rogers, C. B. (2002). *Phytochemistry*, **61**, 955–957. 10.1016/s0031-9422(02)00439-9 12453524

[bb14] Yoganathan, K., Rossant, C., Ng, S., Huang, Y., Butler, M. S. & Buss, A. D. (2003). *J. Nat. Prod.* **66**, 1116–1117. 10.1021/np030146m 12932138

